# Urgent Revascularization of the Left Proximal Circumflex Following Cessation of Cangrelor Within Six Hours of Procedure

**DOI:** 10.7759/cureus.40314

**Published:** 2023-06-12

**Authors:** Sherri Huang, Camilo Rodriguez, Khalid Shakfeh, Jorden Smith, Koushik Reddy

**Affiliations:** 1 Internal Medicine and Pediatrics, University of South Florida Morsani College of Medicine, Tampa, USA; 2 Internal Medicine, University of South Florida Morsani College of Medicine, Tampa, USA; 3 Internal Medicine, University of South Florida Morsani College, Tampa, USA; 4 Cardiology and Lifestyle Medicine, James A. Haley Veterans Affair Medical Center, Tampa, USA

**Keywords:** hypercoagulable state, antiplatelet therapy, thrombotic events, cangrelor, bridging antithrombotic therapy

## Abstract

Patients undergoing procedures are often transitioned off anticoagulants using anti-platelet agents with short half-lives as a "bridge." We present the case of a patient with a history of in-stent thromboses who experienced a thrombotic event following a literature-guided bridging protocol. This case is one of the first to show that stopping cangrelor within six hours led to a need for urgent revascularization and suggests that the timing for discontinuing bridging agents should be customized based on the patient's history of increased blood clotting.

## Introduction

Patients with a history of the acute coronary syndrome (ACS), coronary artery disease (CAD), or percutaneous coronary intervention (PCI) are recommended to take aspirin in conjunction with antiplatelet therapy such as clopidogrel or prasugrel. Depending on the clinical scenario and disease severity, patients who have both ACS and/or a history of PCI and atrial fibrillation may also be advised to take antiplatelet therapy together with anticoagulation such as heparin, warfarin, or direct-acting oral anticoagulants (DOACs) [1,2]. This could also be the case for patients with hypercoagulable states like antiphospholipid syndrome.

Patients taking anti-thrombotic therapy, i.e., anti-platelets or anti-coagulants, are at risk for clotting on abrupt discontinuation of their regimen. When considering the peri-operative management of anti-thrombotic therapy, there must be a careful balance between the thrombotic and bleeding risks [3]. As such, anti-thrombotic agents with short half-lives are often used to "bridge" patients off warfarin, prasugrel, and other anti-thrombotic therapies before procedures. Most research on bridging recommendations is based on warfarin, which is recommended to be discontinued five days before surgery, with the eventual start of low molecular weight heparin (LMWH) or unfractionated heparin three days before surgery. LMWH can be discontinued 24 hours before surgery, while unfractionated heparin can be discontinued four to six hours before the procedure [4].

Currently, there are insufficient clinical trials supporting a clear consensus for bridging anti-platelet therapy in patients with complex thrombotic and bleeding risks [5,6]. In one study evaluating 67 pre-operative bridging agents, glycoprotein IIB/III inhibitors were used as the pre-operative bridging agent for around seven days and stopped four to six hours before surgery. Despite this, two patients suffered from ACS post-operatively, showing that post-operative stent thrombosis can still occur [7]. Similar outcomes were also observed in a meta-analysis with 280 patients who were trialed with pre-operative bridging with a glycoprotein IIB/III inhibitor, showing that with this method, there is still a risk of stent thrombosis and bleeding [8].

Cangrelor is an analogue of adenosine triphosphate that acts on the P2Y12 receptor, exhibiting a dose-dependent, linear inhibition of platelet aggregation [9]. Several studies have evaluated the use of shorter-acting antiplatelet agents such as eptifibatide or cangrelor as bridging therapy, whereupon the P2Y12 inhibitor would be stopped five to seven days prior to surgery and then transitioned to the shorter-acting agents and continued until an hour to six hours prior to surgery [7,8,10]. A trial showed that cangrelor can maintain a low risk of thrombosis while also avoiding the risk of bleeding [10]. However, further research is warranted to clarify bridging guidelines for anti-platelet therapy in patients with complex medical histories so that clinicians can effectively balance the risks of thrombosis and bleeding in these patients. Moreover, research should account for specific cases of patients with a history of hypercoagulability. Here, we present a case in which a patient experiences a clotting event when bridged off his prasugrel, despite following a literature-guided bridging protocol.

This article was previously presented as a short oral presentation and e-poster on June 28th, 2022, at the 2022 CHEST Congress in Bologna, Italy.

## Case presentation

Our patient is a 60-year-old male with a past medical history of serum positivity for lupus anticoagulants, CAD status post-three-vessel coronary artery bypass surgery (CABG) with recurrent thromboses, and a history of 11 stents with in-stent thromboses while on clopidogrel. For his history of hyper-coagulability, at the time of this case, the patient was on prasugrel and rivaroxaban. He presented for bridging off his prasugrel in anticipation of a colonoscopy. The original plan was to bridge with the glycoprotein IIb/IIIa inhibitor eptifibatide, which has a short half-life, and he was admitted to the cardiology service for monitoring during the bridge. On admission, he presented as afebrile and hemodynamically stable, with an unremarkable physical examination. Labs including the complete blood count and basic metabolic panel were unremarkable; the platelet count was 204 and the INR was 1.08.

He was transitioned to eptifibatide a day after discontinuing his prasugrel. However, due to worsening thrombocytopenia, eptifibatide was discontinued two days following initiation, at which point cangrelor was initiated. Rivaroxaban was discontinued three days prior to the colonoscopy. Using data from the literature that showed that cangrelor can be discontinued six hours after the procedure, the patient's cangrelor was discontinued two hours prior to his colonoscopy [10].

**Figure 1:**
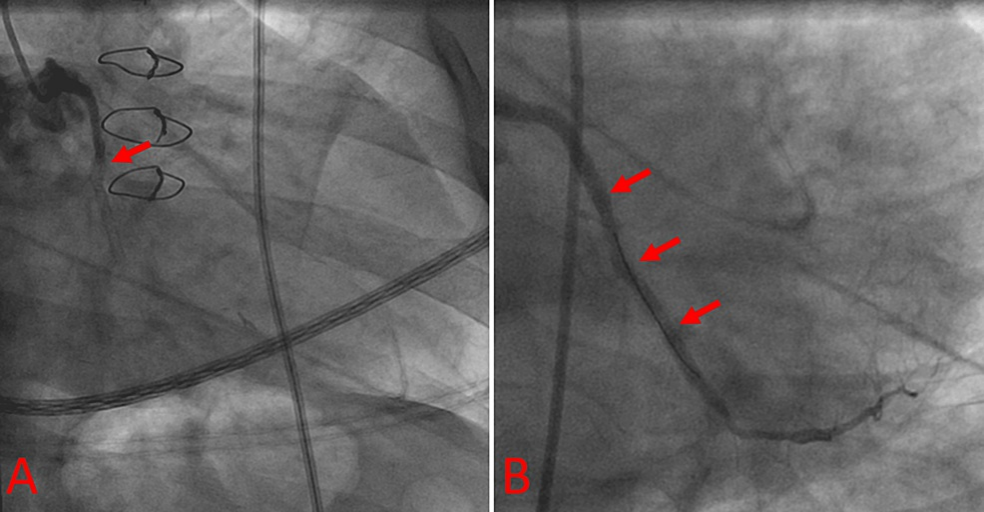
Occlusion and revascularization of the proximal left circumflex in the case patient. (A) Occlusion of the proximal left circumflex; arrow indicates site of dye flow obstruction during the left heart catheterization. (B) Following percutaneous old balloon angioplasty, arrows indicate continuous dye flow representing successful revascularization.

Within two hours, the patient began experiencing sudden, crushing chest pain. EKG and troponin I were ordered stat. He was rushed to the catheterization lab, during which he was found to have an acute total occlusion of his proximal left circumflex, for which he underwent a percutaneous old balloon angioplasty (Figure 1A-1B). He was loaded with prasugrel in the catheterization lab, started on dual antiplatelet therapy (DAPT) with the addition of aspirin (81 mg daily), and re-started on his rivaroxaban.

He underwent an echocardiogram, which showed an ejection fraction (EF) of 50-55%, inferolateral and inferior wall hypokinesis, moderate aortic regurgitation, mild tricuspid regurgitation, and a right ventricular systolic pressure of 44 mmHg. The patient was advised to stay inpatient to be monitored for at least another day post-angioplasty and following the resumption of his anticoagulation. In addition, the hematology service was consulted to evaluate the patient for optimization of anticoagulation therapy given his CAD, lupus anticoagulant-positive status, and hypercoagulability history. However, the patient insisted on being discharged. He was chest pain-free and hemodynamically stable. He was discharged on DAPT and the same rivaroxaban regimen.

## Discussion

Abrupt discontinuation of anti-platelet therapy carries a risk of ischemic complications. Consequently, platelet inhibitors that continue anti-coagulation and whose effects are quickly reversed on agent cessation are desirable as "bridges" prior to surgeries and other procedures with a risk of bleeding. The pharmacokinetic profiles of eptifibatide and cangrelor allow for a quick reversal of anti-coagulation. Different bridging strategies include initiating bridging with either a glycoprotein IIb/IIIa antagonist or cangrelor. In the case of our patient, the initial strategy to bridge with eptifibatide and the timeline of bridging are based off current guidelines, which employ several clinical concepts. First, in the prevention of coronary arterial thromboses, anti-platelet agents are preferred over anticoagulants as platelet aggregation is a key step in the formation of arterial thromboses [11]. Glycoprotein IIb/IIIa antagonists are also short- and fast-acting, making them ideal for the fast onset and offset of bridging. Moreover, the choice for eptifibatide is based on data on its safety and ease of administration. For example, in a study of 100 patients with implanted coronary stents who were bridged to the surgery off thienopyridine therapy with eptifibatide, there was no difference in bleeding outcomes as evaluated by the need for blood transfusions between the eptifibatide-bridged group and the control group [12]. As per guidelines, prasugrel, which has a higher risk of bleeding compared to clopidogrel in several studies, was discontinued a week prior to the procedure [13].

Thrombocytopenia is a known side effect of glycoprotein IIb/IIIa antagonists and limited the continued use of eptifibatide in the presented case [14]. Cangrelor is a relatively newer agent whose fast onset and offset also allow for a quick reversal of anti-coagulation. Indeed, one pharmacokinetic feature of cangrelor is its faster offset compared to glycoprotein IIb/IIIa antagonists, allowing longer continuous infusions, whereas the "bleeding risk window" of glycoprotein IIb/IIIa antagonists is narrower in comparison [9]. Angiolillo et al. showed that cangreglor maintained platelet inhibition at a higher rate compared to placebo [10]. The study parameters were the discontinuation of cangrelor within one to six hours prior to the surgery, with a minimum of 48 hours up to seven days allowed for the duration of cangrelor infusion. Assessed endpoints included myocardial infarction, stroke, or the need for urgent revascularization. Relevant to our case, the study showed that in the 106 patients who received cangrelor, the incidence of these ischemic events was low, at 2.8%; of this, 0.9% required urgent revascularization.

To date, a number of case studies have evaluated the use of cangrelor in bridging for both high-risk and low-risk procedures. The endpoint assessed is usually thrombotic events and/or bleeding, and the studies show that cangrelor was employed and discontinued without causing complications [15-19]. Specifically, Bowman et al. evaluated the use of cangrelor as bridging therapy for surgical procedures in 11 patients with a history of coronary stents, finding that bleeding complications occurred in three of the patients and no in-stent thromboses occurred [15]. A number of case reports evaluated low-risk procedures [16-18]. In one case, a patient on aspirin and ticagrelor following placement of a drug-eluting stent underwent a bronchoscopy, and cangrelor was used as bridging therapy successfully without thrombotic or bleeding complications [17]. Another patient who had received a drug-eluting stent six weeks prior to obtaining an esophageal balloon dilatation was also bridged successfully with cangrelor [18]. Hu et al. reported the use of cangrelor as bridging therapy for high-risk cervical spine surgery in a patient who had obtained a drug-eluting stent four months prior to the procedure and again demonstrated successful bridging without complications [19].

In a study of 24 patients identified as high-risk for in-stent thromboses and needing non-deferrable surgery, one patient experienced an ST elevation myocardial infarction (STEMI) three hours after cangrelor discontinuation [20]. In this study, thrombotic risk stratification was assessed by medical history, including previous complex PCI with multiple stent placements and left main coronary involvement, PCI within one month, or ACS within three months. In particular, the fatal MI after cangrelor discontinuation occurred in a 70-year-old female who had a history of chronic limb-threatening ischemia and complex PCI with multiple stents and was on aspirin and ticagrelor pre-operatively. She had been scheduled for a non-deferrable lower limb amputation, and her ticagrelor was discontinued five days prior to surgery. The study initiated cangrelor two to three days following ticagrelor discontinuation and discontinued it one to 10 hours prior to the surgery [20]. No other ischemic events occurred pre-operatively or up to 48 hours after surgery. Our case is therefore one of the first to show that cessation of cangrelor within six hours resulted in the need for urgent revascularization. The patient presented here had comorbidities, including lupus coagulant positivity and numerous stents, which likely increased his risk for an ischemic event. These observations indicate that the cessation timeframe for bridging agents should be tailored depending on the patient's history of coagulability.

## Conclusions

Herein, we demonstrate the importance of proper anti-coagulation strategies prior to a procedure in high-risk patients with a history of hypercoagulability. This patient’s case is highly unique given his in-stent thrombosis, despite the typical bridging protocol with cangrelor for high-risk patients. Our case informs the currently limited literature on patients warranting complex management of bleeding and thrombotic risks and emphasizes the need for ongoing re-assessment of similar cases. Prior to a clear consensus, expert opinion and case-by-case evaluation are critical for bridging protocols for patients with a history of hypercoagulability.
